# A facile chemical conversion synthesis of Sb_2_S_3 _nanotubes and the visible light-driven photocatalytic activities

**DOI:** 10.1186/1556-276X-7-199

**Published:** 2012-03-26

**Authors:** Xuemin Shuai, Wenzhong Shen

**Affiliations:** 1 Laboratory of Condensed Matter Spectroscopy and Opto-Electronic Physics, Department of Physics, Shanghai Jiao Tong University, 800 Dong Chuan Road, Shanghai 200240, China; 2 Key Laboratory of Artificial Structures and Quantum Control (Ministry of Education), Department of Physic, Shanghai Jiao Tong University, 800 Dong Chuan Road, Shanghai 200240, China

**Keywords:** Nanotubes, Chemical transformation, Cation exchange, Growth mechanism, Optical and photocatalytic properties

## Abstract

We report a simple chemical conversion and cation exchange technique to realize the synthesis of Sb_2_S_3 _nanotubes at a low temperature of 90°C. The successful chemical conversion from ZnS nanotubes to Sb_2_S_3 _ones benefits from the large difference in solubility between ZnS and Sb_2_S_3_. The as-grown Sb_2_S_3 _nanotubes have been transformed from a weak crystallization to a polycrystalline structure via successive annealing. In addition to the detailed structural, morphological, and optical investigation of the yielded Sb_2_S_3 _nanotubes before and after annealing, we have shown high photocatalytic activities of Sb_2_S_3 _nanotubes for methyl orange degradation under visible light irradiation. This approach offers an effective control of the composition and structure of Sb_2_S_3 _nanomaterials, facilitates the production at a relatively low reaction temperature without the need of organics, templates, or crystal seeds, and can be extended to the synthesis of hollow structures with various compositions and shapes for unique properties.

## Background

Since the discovery of carbon nanotubes in 1991 [1], extensive research has been carried out on one-dimensional (1D) tubular nanostructures, owing to their unique size-dependent properties and remarkable potential applications in electronics, optoelectronics, catalysis, biotechnology, separation, and so on [2-7]. However, the preparation of nanotubes is relatively difficult, and fewer synthetic techniques have been developed compared to those for other 1D nanostructures, such as nanorods and nanowires [8-10]. So far, different types of nanotubes have been prepared by various approaches including vapor-liquid-solid, chemical vapor deposition, template-directed synthesis, and low-dimensional sacrificial precursors [11-14]. Nevertheless, these strategies often require high temperature, special conditions, and tedious procedures, and most of them are complicated and uncontrollable. Therefore, development of a facile, versatile, and effective synthetic pathway to prepare 1D nanotubes is very important and quite necessary. In particular, it is highly desirable to control and manipulate the chemical compositions and structures of nanotubes.

In fact, chemical conversion and cation exchange have been demonstrated as powerful tools to convert the chemical compositions of nanostructures without destroying the original morphology [15,16]. Our previous studies on the transformation of composition in the core/shell microspheres (from ZnO/ZnS to ZnO/Ag_2_S and ZnO/CuS) [17] and in the hollow microspheres, as well as nanotubes (from ZnS to other various metal sulfides) [18,19], have indicated the significance of chemical conversion and cation exchange. Compared to other strategies, the chemical conversion and cation exchange have the following advantages: (1) reactions can take place in a solution under mild conditions (low growth temperature, without any special equipments or templates); (2) this approach is a typical one-step process, which needs no tedious procedures or further purification of the products; (3) the products can be produced on a large scale; and (4) this strategy can be developed as a general method to fabricate functional semiconductor hollow structures with various compositions and shapes for unique properties, which is quite important with respect to technical applications.

As an important V-VI group binary chalcogenide, antimony trisulfide (Sb_2_S_3_) with an energy bandgap varying between 1.5 and 2.2 eV has attracted particular attention, owing to its good photovoltaic properties, high thermoelectric power [20], broad spectrum response, and suitable valence band position [21]. This material has been applied in various areas such as television cameras with photoconducting targets, thermoelectric cooling devices, electronic and optoelectronic devices, solar energy conversion, and visible light-responsive photocatalysis [20-26]. It has been demonstrated that the properties of antimony trisulfide are determined predominantly by their crystal structure, size, and morphology. Therefore, the synthesis of Sb_2_S_3 _materials with well-controlled size and shape is of great significance for their applications. Up to date, a variety of 1D nanostructures of Sb_2_S_3 _such as nanorods [27-30], nanowires [31], microtubes [32,33], and nanoribbons [34] have already been synthesized by various methods. Nevertheless, little has been devoted to the development of a general and low-cost synthetic method to fabricate Sb_2_S_3 _nanotubes without using any templates or crystal seeds. Although as-grown Sb_2_S_3 _presents in general an amorphous structure, it can be transformed in the polycrystalline phase by successive annealing [35]. Considering the technical importance of this material, fabrication of Sb_2_S_3 _with some inspired structures such as a tubular structure by a convenient and efficient method has always been a great interest.

In this paper, we have realized the first synthesis of Sb_2_S_3 _nanotubes by conversion from ZnS nanotubes via chemical conversion and cation exchange at a low temperature of 90°C. The key point of the method is to utilize the large difference in solubility between ZnS and Sb_2_S_3 _for the effective transformation. Structural, morphological, and optical changes have been observed in these samples after annealing at different temperatures in an argon atmosphere. We have further shown high photocatalytic activities of Sb_2_S_3 _nanotubes for methyl orange (MO) degradation under visible light irradiation, due to the large specific surface area and good crystallinity [36,37]. The present technique is very convenient and efficient, free of any organics, templates, or crystal seeds, and has been demonstrated to control and manipulate effectively the chemical compositions and structures of nanotubes.

## Methods

### Synthesis of ZnS nanotubes

The preparation details for ZnS nanotubes can be found in our recently published papers [19]. Briefly, ZnO nanowires were first prepared by a hydrothermal process. As a typical synthesis process, 0.2 g ZnCl_2 _and 20.0 g Na_2_CO_3 _were added into a 50-mL Telfon-lined stainless steel autoclave and filled with distilled water up to 90% of its volume. After vigorous stirring for 30 min, the autoclave was maintained at 140°C for 12 h, followed by cooling down naturally to room temperature. The synthesis of ZnO nanowires could be realized after the product was washed and dried. Subsequently, the as-prepared ZnO nanowires on substrates (silicon or glass slides) were transferred to a Pyrex glass bottle containing 40 mL of 0.2 M thioacetamide (TAA). The sealed bottle was then heated to 90°C for 9 h in a conventional laboratory oven to synthesize ZnS nanotubes. The final products on the substrates were washed repeatedly with deionized water and then dried at 60°C before being used for the next step in the reaction and further characterization.

### Synthesis of Sb_2_S_3 _nanotubes

The synthesis of Sb_2_S_3 _nanotubes was realized by transferring the silicon or glass slides with ZnS nanotubes on them to a Pyrex glass bottle containing 150 mM C_8_H_4_K_2_O_12_Sb_2 _and 70 mM tartaric acid. During the reaction process, the solution temperature was kept at 90°C. The final products on the substrates were washed thoroughly using deionized water to remove any co-precipitated salts and then dried at air at 60°C. For better crystal quality, the as-prepared Sb_2_S_3 _nanotubes were annealed in an argon atmosphere.

### Morphological and structural characterization

The morphology and structure of the samples were characterized using field-emission scanning electron microscopy (FE-SEM; Philips XL30FEG, FEI Co., Hillsboro, OR, USA) with an accelerating voltage of 5 kV and a high-resolution transmission electron microscopy (HRTEM; JEOL JEM-2100 F, JEOL Ltd., Akishima, Tokyo, Japan). Selected area electron diffraction (SAED) and energy dispersive X-ray (EDX) microanalysis were also performed during the TEM and SEM observations. X-ray diffraction (XRD) was carried out on a diffractometer (D/max-2200/PC, Rigaku Corporation, Tokyo, Japan) equipped with a high intensity Cu Kα radiation (*λ *= 1.5418 Å). Raman spectra were measured at room temperature on a Jobin Yvon LabRAM HR 800 UV micro-Raman/PL system (HORIBA Jobin Yvon Inc., Edison, NJ, USA)at a backscattering configuration under the excitation of a He-Cd laser (325.0 nm) for ZnS nanotubes and a Ar^+ ^laser (514.5 nm) for Sb_2_S_3 _nanotubes.

### Photocatalytic activity measurements

The photocatalytic activities under visible light were monitored through the photodegradation of MO. Visible light irradiation was carried out using a 500-W Xe lamp with a 420-nm UV cutoff filter, which was surrounded by a quartz jacket to allow for water cooling. Photocatalyst powder (30 mg) was added into 80 mL of aqueous MO (20 mg L^-1^) solution and magnetically stirred in the dark for 30 min to reach the adsorption-desorption equilibrium before visible light illumination. The absorbance of the corresponding target organics was monitored by measuring with a UV-vis spectrophotometer (PerkinElmer Lambda 950, PerkinElmer, Waltham, MA, USA).

## Results and discussions

**Figure 1:**
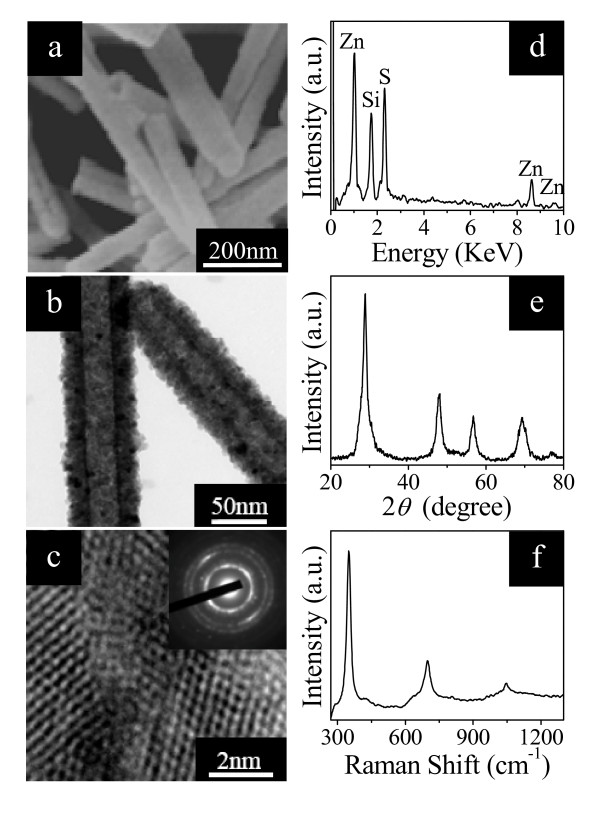
**Images of ZnS nanotubes with their corresponding spectra**. (**a**) FE-SEM and (**b**) TEM images of ZnS nanotubes. (**c**) HRTEM image of a ZnS nanotube shell, together with the corresponding SAED pattern shown in the inset. The corresponding (**d**) EDX, (**e**) XRD, and (**f**) room-temperature Raman spectra of ZnS nanotubes.

In our experiments, we start from the ZnO nanowires which were prepared by a hydrothermal process [19]. We then transfer the ZnO nanowires into a solution containing 0.2 M TAA to convert the ZnO nanowires into ZnS nanotubes. The TAA solution provides sulfide ions to react with zinc ions dissolved from the ZnO nanowires to form ZnO/ZnS core/shell structures. When prolonging the sulfidation time to 9 h under hydrothermal conditions, all ZnO nanowires can change into ZnS nanotubes due to the Kirkendall effect, which normally refers to comparative diffusive migrations among different atomic species in metals and/or alloys under thermally activated conditions [38]. Figure 1a shows the FE-SEM image of the obtained ZnS nanotubes. One can see that some of the shells have an irregular open tip, demonstrating the hollow nature of the prepared nanotubes. Further evidence for the hollow structure can be found from the TEM observation. Figure 1b displays the TEM image of the obtained ZnS nanotubes. The strong contrast difference in the nanotubes with a light inner center and a relatively dark edge confirms that the yielded ZnS nanotubes are all hollow. Figure 1c presents a HRTEM image taken on the edge of the ZnS nanotube, which clearly exhibits that the shell is composed of ZnS nanocrystalline grains with a polycrystalline nature. The inset of Figure 1c is the corresponding ring-like SAED pattern without a spotted pattern taken on a single nanotube, also providing evidence for the polycrystalline nature of ZnS nanotubes. The composition of the ZnS nanotubes can be easily identified by the EDX (Figure 1d) and XRD (Figure 1e) spectra. Figure 1f shows the room-temperature Raman spectrum of the ZnS nanotubes. The observation of multiple resonant Raman peaks indicates that the yielded ZnS nanotubes possess good optical quality [39].

**Figure 2:**
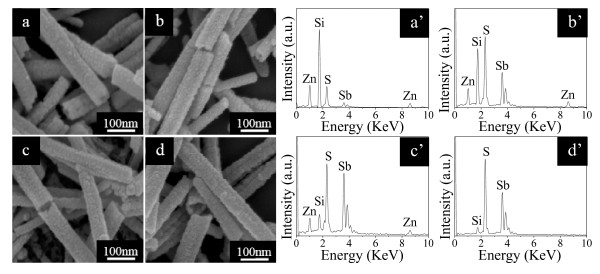
**FE-SEM images of Sb_2_S_3 _nanotubes and their corresponding EDX spectra with different reaction times**. FE-SEM images of Sb_2_S_3 _nanotubes with different reaction times (**a**) 1 h, (**b**) 3 h, (**c**) 8 h, and (**d**) 16 h. (**a' **to **d'**) The corresponding EDX of Sb_2_S_3 _nanotubes with different reaction times.

The main attempts in the present work are to synthesize Sb_2_S_3 _nanotubes and to investigate their optical properties and photocatalytic performances. To make the conversion of ZnS nanotubes to Sb_2_S_3 _ones, we transfer the substrates with ZnS nanotubes on them into 40 mL of 150 mM C_8_H_4_K_2_O_12_Sb_2 _and 70 mM tartaric acid aqueous solution. A series of time-dependent experiments were conducted to track the formation process of Sb_2_S_3 _tubular structures, as shown in Figure 2. Under the reaction time of 1 h, some Sb_2_S_3 _nanoparticles on the ZnS nanotubes were observed because ion exchange happens as Sb^3+ ^reacts with S^2- ^slowly dissolved from the surface of ZnS nanotubes to form initial Sb_2_S_3 _shells, as depicted in Figure 2a. After another 2-h reaction, more Sb_2_S_3 _nanoparticles piled up on the initial Sb_2_S_3 _shells (Figure 2b). When the reaction time reached to 8 h, large numbers of Sb_2_S_3 _nanoparticles were produced (Figure 2c). When further prolonging the reaction time to 16 h, uniform Sb_2_S_3 _nanotubes of large quantities with diameters of about 70 nm were fully converted from ZnS ones (Figure 2d).

The corresponding EDX spectra in Figures 2a',b',c',d' give clear evidence for the FE-SEM observation of the samples obtained through various reaction times. From Figure 2a', we can observe the successful incorporation of the Sb element into the ZnS nanotubes in the compositional information. The signal of Si originates from the substrate. With the increase of the reaction time, the Sb/Zn stoichiometric ratio becomes higher and higher due to the fact that more and more Zn atoms were replaced by Sb atoms with the reaction processing, as shown in Figures 2b',c'. Further chemical reaction will yield pure Sb_2_S_3 _nanotubes, which can be unambiguously confirmed by the EDX spectrum in Figure 2d'. There are only Sb, S, and Si elements without any Zn element.

According to the experimental observation described above, the whole process can be described as follows: Once the obtained ZnS nanotubes were transferred into C_8_H_4_K_2_O_12_Sb_2 _solution, cation exchange began at the interfaces between the ZnS nanotube surfaces and solution. With the increase in the reaction time, Zn^2+ ^was gradually substituted by Sb^3+^, resulting in the synthesis of Sb_2_S_3 _nanotubes. The driving force for the cation exchange is provided by the large difference in solubility between ZnS and Sb_2_S_3 _(solubility product constant (*K*_sp_) of ZnS is 2.93 × 10^-25^, whereas *K*_sp _of Sb_2_S_3 _is 1.5 × 10^-93^) [40]. The above conversion mechanism reveals that the ZnS nanotubes can act as both reactants and templates during the cation-exchange process. Therefore, a general, facile, and economic method has been proposed and realized to synthesize Sb_2_S_3 _nanotubes, and this strategy can control and manipulate effectively the chemical compositions and structures of nanotubes. Furthermore, we can extend this chemical conversion approach to the synthesis of other metal sulfide nanotubes under the condition that those yielded metal sulfides have lower *K*_sp _values than ZnS. In fact, it is because of the large *K*_sp _in ZnS that we choose ZnS nanotubes as the reactants and templates to synthesize various metal sulfide nanotubes, like Ag_2_S, CuS, PbS, Bi_2_S_3 _[19], and Sb_2_S_3 _nanotubes in the present paper. It is a convenient one-pot method without using any organics, templates, or crystal seeds and has great potential in industrialized high-volume production.

**Figure 3:**
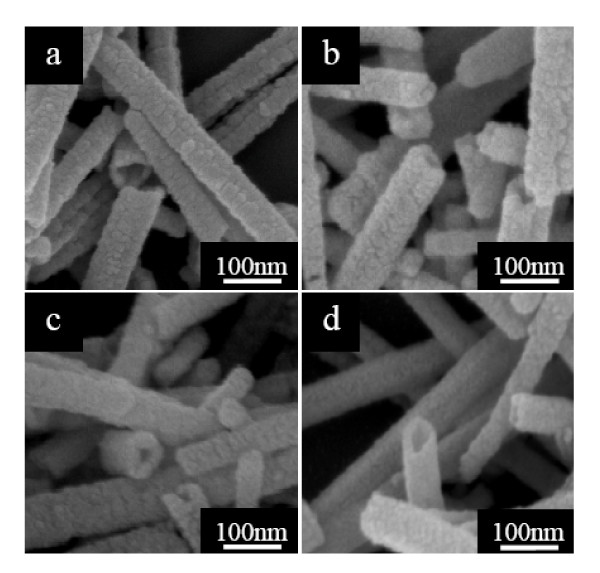
**FE-SEM images of the Sb_2_S_3 _nanotubes under different annealing temperatures**. (**a**) as-prepared at 90°C for 16 h before annealing; annealed in argon atmosphere at (**b**) 200°C, (**c**) 250°C, and (**d**) 400°C for 1 h, respectively.

The annealing treatment exerts an important influence on the morphology and structure of the Sb_2_S_3 _nanotubes. Figure 3a presents the SEM image of Sb_2_S_3 _nanotubes fabricated at 90°C for 16 h before annealing, clearly showing that these nanotubes exhibit rough structures with myriad Sb_2_S_3 _nanoparticles. When the as-prepared Sb_2_S_3 _16-h nanotubes were annealed in argon atmosphere under 200°C for 1 h, the nanotubes were gained with Sb_2_S_3 _nanoparticles agglomerating on the surface (Figure 3b), and compact and uniform nanotubes were observed for Sb_2_S_3 _16-h nanotubes annealed at higher temperature of 250°C (Figure 3c). Further increasing the annealing temperature to 400°C, we were able to realize more uniform and slippery Sb_2_S_3 _nanotubes, as illustrated in Figure 3d.

**Figure 4:**
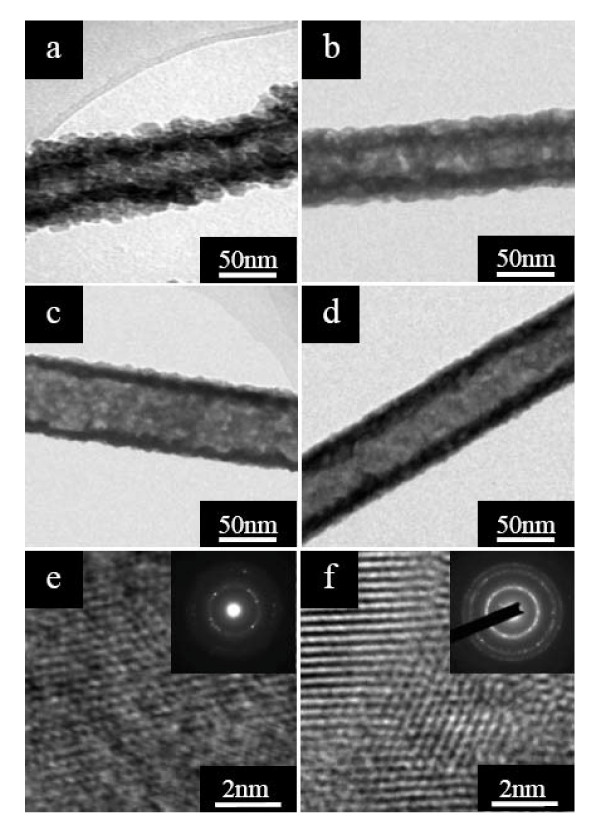
**TEM and HRTEM images of Sb_2_S_3 _16-h nanotubes**. TEM images of the Sb_2_S_3 _16-h nanotubes (**a**) just grown and annealed in argon at (**b**) 200°C, (**c**) 250°C, and (**d**) 400°C for 1 h. HRTEM images of the Sb_2_S_3 _16-h nanotubes (**e**) just grown and (**f**) annealed at 400°C, together with the corresponding SAED patterns shown in the insets.

We have investigated the crystal structures of the Sb_2_S_3 _nanotubes under different annealing temperatures by TEM and HRTEM. Figure 4a shows the TEM image of as-prepared Sb_2_S_3 _nanotubes obtained at 16 h before annealing. One can notice that the outer layers were composed of numerous Sb_2_S_3 _nanoparticles with a mean size of 18 nm. As the Sb_2_S_3 _16-h nanotubes were annealed in argon atmosphere at 200°C for 1 h, the Sb_2_S_3 _nanoparticles on the surface of nanotubes became coacervated (Figure 4b), and compact and uniform nanotubes were formed at a higher annealing temperature of 250°C (Figure 4c). Figure 4d presents the TEM image of the Sb_2_S_3 _16-h nanotubes with the annealing temperature increased to 400°C, where the Sb_2_S_3 _16-h nanotubes appear to be smooth on the surface, and the diameter of the nanotubes is about 70 nm with a shell as thick as 18 to 21 nm.

HRTEM observation can give deep insight into the structural features of the Sb_2_S_3 _nanotubes before and after annealing. Figure 4e is a representative HRTEM image taken on the edge of the obtained Sb_2_S_3 _16-h nanotube before annealing (Figure 4a). The lattice fringes are highly disordered and ambiguous, revealing that the un-annealed Sb_2_S_3 _16-h nanotubes have poor crystallization [34]. The corresponding SAED pattern of the nanotube (inset of Figure 4e) exhibits weak ring diffractions, indicating slight crystallization. Figure 4f presents a HRTEM image recorded from a certain Sb_2_S_3 _16-h nanotube after annealing at 400°C (Figure 4d); only the polycrystalline nature of Sb_2_S_3 _nanotubes can be observed. The clearly observed crystal lattice fringes demonstrate that the nanotubes are highly crystallized and free from dislocation and stacking faults [24]. The corresponding SAED pattern shown in the inset of Figure 4f having characteristic ring diffractions also confirms the polycrystalline feature of the nanotubes after annealing.

**Figure 5:**
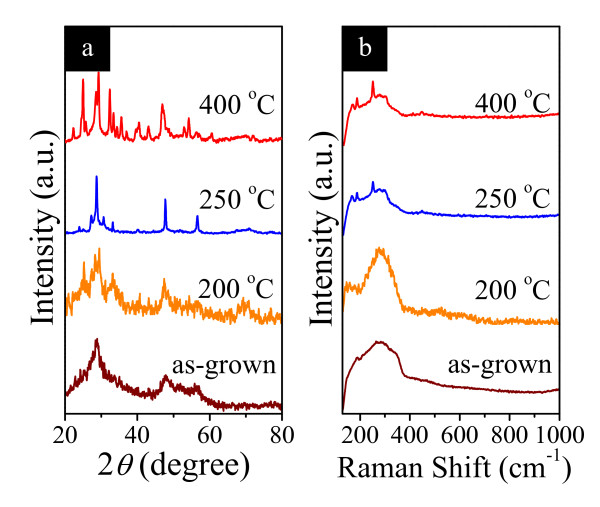
**XRD patterns and Raman spectra**. (**a**) XRD patterns and (**b**) room-temperature Raman spectra of the Sb_2_S_3 _16-h nanotubes just grown and annealed in argon at 200°C, 250°C, and 400°C for 1 h.

The effect of argon annealing treatment on the crystallographic properties of Sb_2_S_3 _nanotubes has been further revealed by the XRD patterns for Sb_2_S_3 _16-h nanotubes annealed at different temperatures. As shown in Figure 5a, for the sample before annealing, the broadening and low intensity of the diffraction peaks indicate weak crystallization of the sample [37]. When annealed at 200°C for 1 h, indistinct diffraction peaks of the sample appeared. Peaks become sharper as the annealing temperature increases to 250°C, as can be seen in the same figure, while the intensity and shape of the diffraction peaks reveal that the sample is not perfectly crystallized [41]. Upon increasing the annealing temperature to 400°C, the peak intensities steadily become stronger, showing an enhancement of the crystallization. All of the clear diffraction peaks can be indexed to an orthorhombic phase of Sb_2_S_3 _(JCPDS Files, No. 06-0474). The shape of the diffraction peaks demonstrates that the products should be well crystallized [41]. No other impurities were found in the samples, indicating that the products are pure stibnite Sb_2_S_3_.

To confirm the transition from a weak crystallization to a polycrystalline structure, Raman spectra have also been measured at different annealing temperatures. The results are summarized in Figure 5b. For Sb_2_S_3 _16-h nanotubes before annealing, the spectrum is very broad, indicating poor crystallinity [35]. The sample after annealing in argon at 200°C for 1 h presents similar spectrum to the un-annealed sample but with a single peak. At higher temperatures of 250°C and 400°C, several sharp peaks appear, which correspond to the Raman spectra of crystalline Sb_2_S_3 _(stibnite structure) [24,42]. The band centered at 170 cm^-1 ^can be assigned to the vibration of Sb-Sb bonds in S_2_Sb-SbS_2 _structural units [43]. The presence of peaks at 189 and 252 cm^-1 ^suggests the formation of a good crystalline product [42]. The peak at 279 cm^-1 ^is in accordance with the symmetric vibrations of SbS_3 _pyramidal units having C_3v _symmetry [44,45], and the peak at 450 cm^-1^, with the S-S vibrations [44] or the symmetric stretching of the Sb-S-S-Sb structural units [45]. These results agree well with the XRD observation in Figure 5a. Our Sb_2_S_3 _nanotubes will yield a poor morphology and crystal quality when annealed above 400°C, which can be attributed to a sulfur deficiency as a consequence of sulfur loss during the high-temperature annealing without sulfur vapor [35].

**Figure 6:**
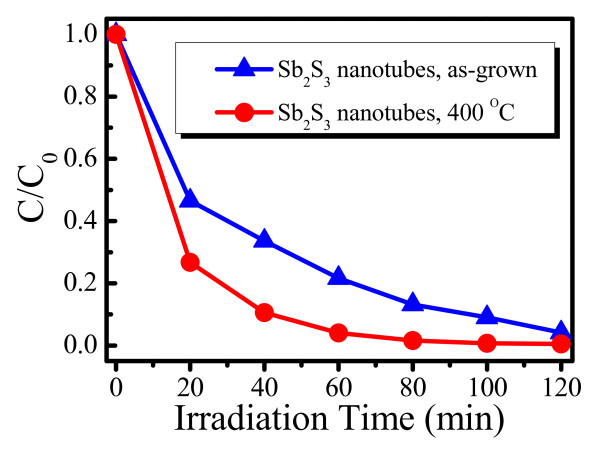
**Visible-light-driven photocatalytic activities of Sb_2_S_3 _16-h nanotubes before and after annealing**. The nanotubes were annealed in argon at 400°C for 1 h.

To characterize the photocatalytic efficiency of Sb_2_S_3 _nanotubes, we employ MO as a model pollutant. Figure 6 shows photocatalytic MO degradation over the Sb_2_S_3 _16-h nanotubes before and after annealing at 400°C under visible light (*C*_0 _and *C *are the equilibrium concentration of MO before and after visible light irradiation, respectively), from which one can see that our as-prepared Sb_2_S_3 _16-h nanotubes show great visible light-induced photocatalytic activities and that the degradation percentage of MO increases rapidly with increasing time. The high photodegradation rate of MO (driven by visible light) can be attributed to the large specific surface area of nanotubes since the enlarged surface helps to increase the photocatalytic reaction sites and promote the efficiency of the electron-hole separation [36]. Furthermore, we are able to achieve significant improvement on the photocatalysis activities in the Sb_2_S_3 _16-h nanotubes calcined in argon at 400°C, and the degradation percentage is nearly complete in a time period of 120 min, which indicates that the crystalline phase should be another main factor influencing the photocatalytic activities. Therefore, the large surface area of the Sb_2_S_3 _nanotubes was not the only factor responsible for the high photocatalytic activities, and the good crystallinity could also be critical, which may be due to the fact that the better the crystallinity, the fewer lattice defects act as recombination centers for photoinduced electrons and holes [37]. As far as we know, the degradation efficiency of our Sb_2_S_3 _nanotubes on MO is comparable with other oxides and sulfides [21,24,25,46].

## Conclusions

In summary, Sb_2_S_3 _nanotubes have been successfully synthesized by chemical conversion and cation exchange at a low temperature of 90°C. The conversion mechanism of the Sb_2_S_3 _nanotubes from ZnS nanotubes is due to the large difference in solubility between ZnS and Sb_2_S_3_. Samples have been annealed at different temperatures in the range of 200°C to 400°C in an argon atmosphere. The morphological, structural, and optical characteristics of the yielded Sb_2_S_3 _nanotubes before and after annealing were characterized by SEM, TEM, XRD, and Raman spectra in detail. It is revealed that the synthesized Sb_2_S_3 _nanotubes can be transformed from a weak crystallization to a polycrystalline structure through the successive annealing treatment. Furthermore, the Sb_2_S_3 _nanotubes exhibit high photocatalytic activities for MO degradation under visible light irradiation as a result of large specific surface area and good crystallinity. The present strategy is a very convenient and efficient method to control and manipulate effectively the chemical composition and structure of nanomaterials. Although the present work focuses on Sb_2_S_3 _nanotubes, other metal sulfide hollow structures are also expected to be realized based on ZnS hollow structures with the corresponding shapes as the precursors during the chemical conversion process. We have therefore expected that the general and economic technique of material synthesis demonstrated in this article can be used in a broad range of applications to fabricate innovative micro- and nanostructured semiconductor materials with different compositions and geometries having unique properties.

## Abbreviations

EDX: energy dispersive X-ray; FE-SEM: field-emission scanning electron microscopy; HRTEM: high-resolution transmission electron microscopy; MO: methyl orange; 1D: one-dimensional; SAED: selected area electron diffraction; TAA: thioacetamide; XRD: X-ray diffraction.

## Competing interests

The authors declare that they have no competing interests.

## Authors' contributions

XS participated in the design of the study, carried out the experiments, and performed the statistical analysis, as well as drafted the manuscript. WS took charge of the design of the study, provided the theoretical and experimental guidance, and revised the manuscript. All authors read and approved the final manuscript.
